# Supplementary material for: Stromal FOXF2 suppresses prostate cancer progression and metastasis by enhancing antitumor immunity
Source: Nat Commun. 2022 Nov 11;13:6828. doi: 10.1038/s41467-022-34665-z (PMC9652358; doi:10.1038/s41467-022-34665-z)
Supplement: Supplementary file 1 — Supplementary Information [file 41467_2022_34665_MOESM1_ESM.pdf]

Supplementary Information

Stromal FOXF2 suppresses prostate cancer progression and metastasis by enhancing antitumor immunity

Deyong Jia[1], Zhicheng Zhou[1], Oh-Joon Kwon[1], Li Zhang[1], Xing Wei[1], Yiqun Zhang[2], Mingyang Yi[3], Martine P Roudier[1], Mary C Regier[4,5], Ruth Dumpit[6], Peter S Nelson[6], Mark Headley[6], Lawrence True[5], Daniel W Lin[1], Colm Morrissey[1], Chad J Creighton[2], Li Xin[1,4,7]

Supplementary Figure 1

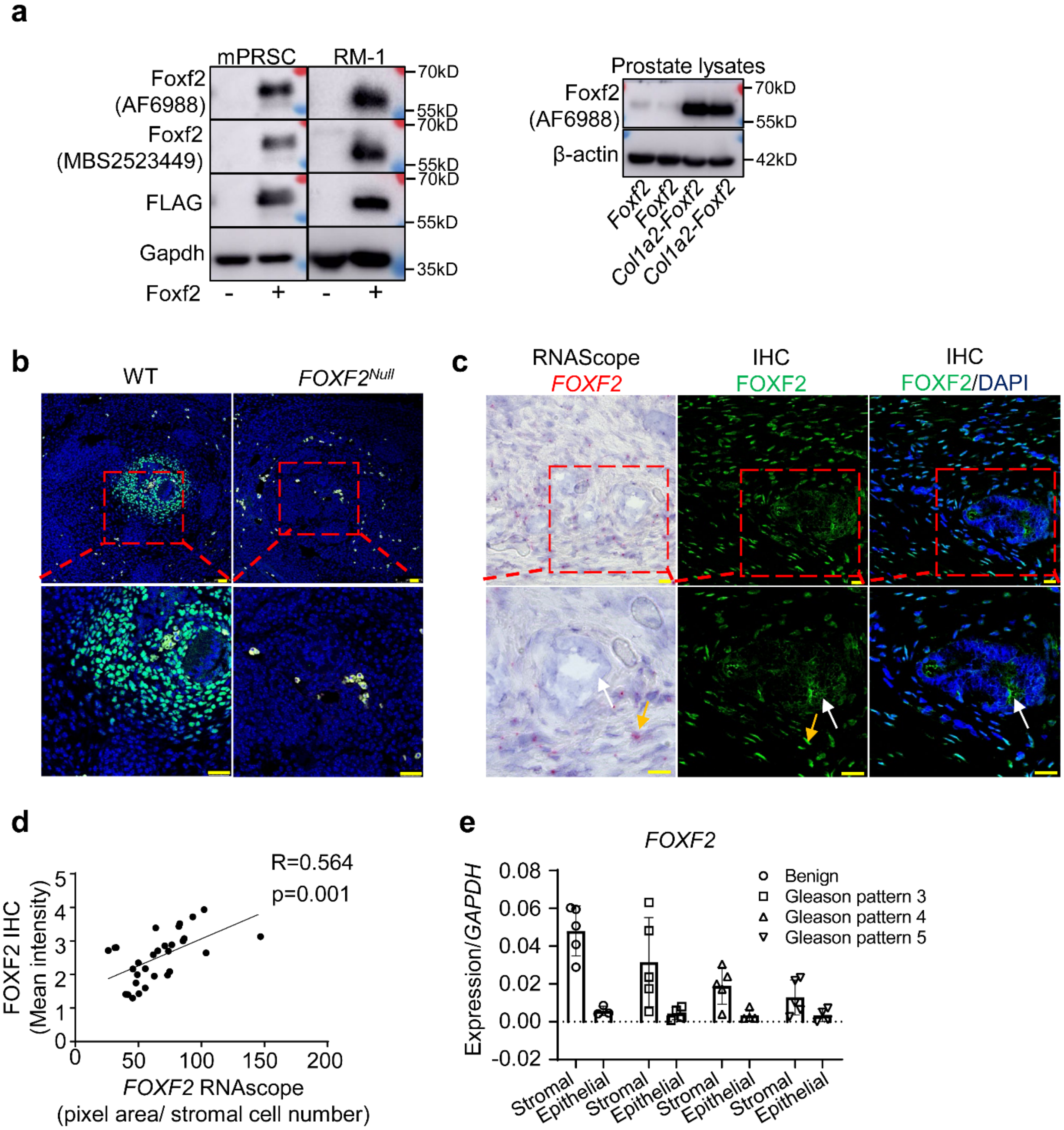

Supplementary Fig. 1: Stromal FOXF2 expression inversely correlates with Gleason patterns.

(a) Western blot analyses of mouse prostate stromal cells and RM-1 cells that ectopically expressed a 3xFLAG-tagged Foxf2 and prostate tissues from tamoxifen-treated *R26-LSL-Foxf2* (*Foxf2*) and *Cola2-CreER^{T2};R26-LSL-Foxf2* (*Col1a2-Foxf2*) mice using sheep (AF6988 from R&D) and rabbit (MBS2523449 from MyBioSource) anti-Foxf2 antibodies. Experiments were repeated three times with similar observation. Source data are provided in Supplementary Fig. 13. (b) Immunostaining of Foxf2 in wild type (WT) and *Foxf2* null embryos. Areas framed by red dotted rectangle show

staining of Foxf2 in eye tissue of WT but not null mouse. Bars = 25 μm. Experiments were repeated twice with similar observation. (c) RNA-in-situ analysis of *FOXF2* in human prostate cancer specimens (Left panels) shows no sign of *FOXF2* expression in tumor cells (white arrow, lower left panel) where immunostaining display unspecific staining by anti-Foxf2 antibody (white arrows, lower middle and lower right panels). Bars = 25 μm. Image represents staining obtained from 1 out of 7 specimens. (d) Dot plot shows positive correlation between intensity of immunostaining of stromal FOXF2 and that of RNA in Situ analysis for *FOXF2* in stromal cells of normal human prostate tissues by Person Correlation analysis. Each dot represents signal intensity of FOXF2 and *FOXF2* collected from the corresponding fields on sequential slides. N=30 region from 7 specimens. (e) qRT-PCR analysis of Foxf2 in laser-captured epithelial (benign and tumor, N=15 regions) and stromal cells (N=20 regions) from a total of 13 benign human prostate tissues and prostate cancer specimens. Data represent means ± s.d.

Supplementary Figure 2

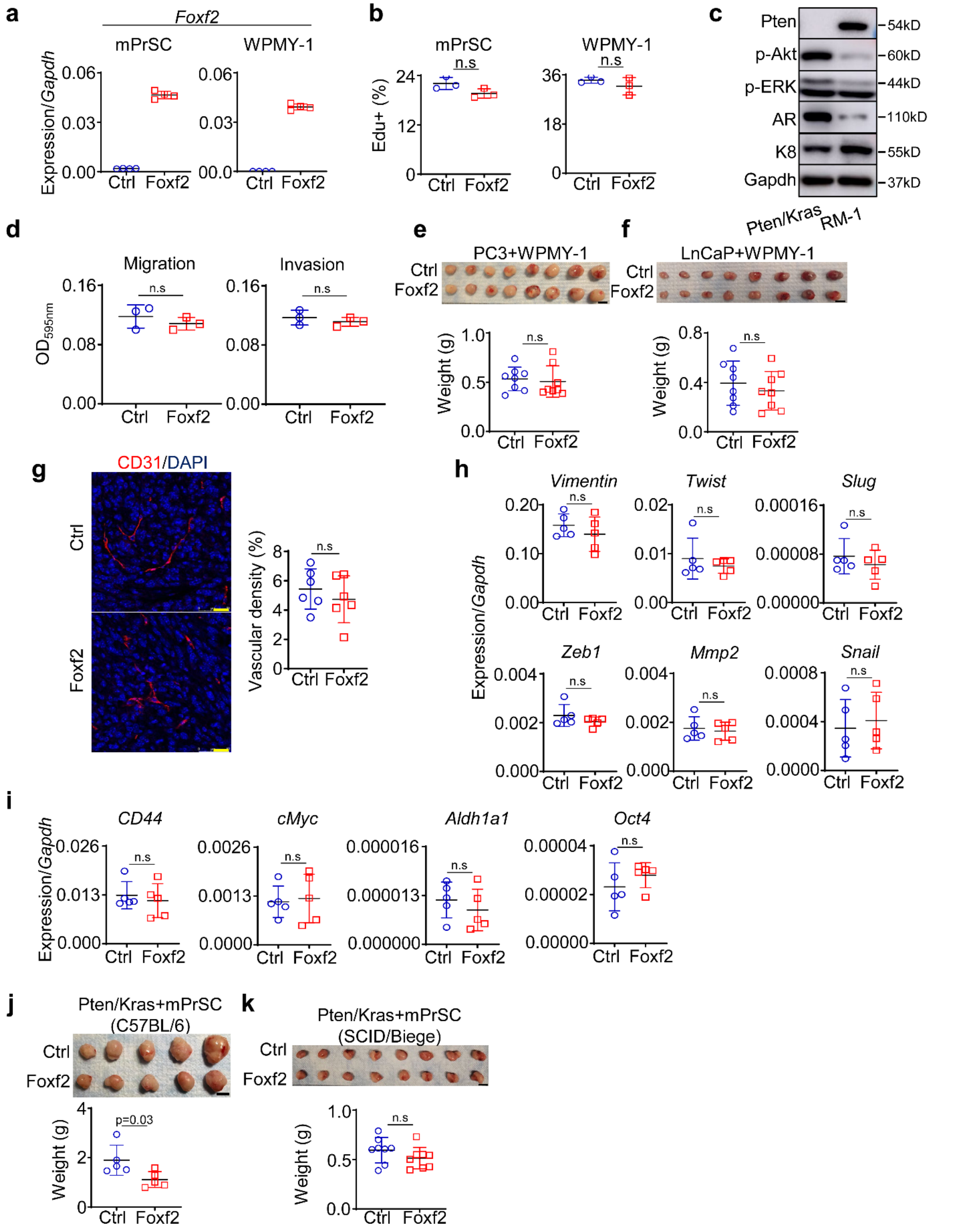

Supplementary Fig. 2: Stromal Foxf2 overexpression does not affect prostate cancer biology *in vitro* or in immunocompromised context *in vivo*.

(a) QRT-PCR of *Foxf2* in control and Foxf2-expressing mouse prostate stromal cells (mPrSC) and WPMY-1 cells. Data represent means ± s.d. from 4 independent experiments. (b) EdU incorporation assay. Data represent means ± s.d. of percentages of EdU^+ cells from 3 independent experiments. Two-sided unpaired t-test. (c) Western blot analysis of RM-1 and Pten/Kras cells. Experiments were repeated for three times with similar pattern. Source data are provided in Supplementary Fig. 13. (d) Boyden chamber assays to determine the capacity of RM-1 cells for migration and invasion when culturing with mouse prostate stromal cells infected with control (Ctrl) and Foxf2-expressing (Foxf2) lentiviruses. OD_{595} values reflect number of cells that have migrated or invaded through chambers. Data represent means ± s.d. from 3 experiments. Two-sided unpaired t-test. (e) Image of subcutaneous tumors grown from PC3 cells cocultured with control and Foxf2-expressing WPMY-1 in SCID/Beige hosts. Bar = 1 cm. Dot plot shows means ± s.d. of tumor weight. N=8 tumor per group. Two-sided unpaired t-test. (f) Image of subcutaneous tumors grown from LnCaP cells cocultured with control and Foxf2-expressing WPMY-1 in SCID/Beige hosts. Bar = 1 cm. Dot plot shows means ± s.d. of tumor weight. N=8 per group. Two-sided unpaired t-test. (g) Immunostaining of CD31 in RM-1 xenografts grown in SCID/Biege hosts with control (Ctrl) and Foxf2-expressing (Foxf2) mouse prostate stromal cells. Dot plot shows means ± s.d. of percentage of CD31 staining pixel per image field. Each dot represents average value of 10 images from each xenograft. Bars= 25μm. N=6 mice per group. Two-sided unpaired t-test. (h) QRT-PCR of EMT-related genes in RM-1 xenografts grown in SCID/Biege hosts with control (Ctr) and Foxf2-expressing (Foxf2) mouse prostate stromal cells. Data represent means ± s.d. from 5 independent xenografts. Two-sided unpaired t-test. (i) QRT-PCR of cancer stem cell-related genes in RM-1 xenografts grown in SCID/Biege hosts with control (Ctrl) and Foxf2-expressing (Foxf2) mouse prostate stromal cells. Data represent means ± s.d. from 5 independent xenografts. Two-sided unpaired t-test. (j) Image of subcutaneous tumors grown from Pten/Kras cells cocultured with control and Foxf2-expressing mouse prostate stromal cells (mPrSC) in male C57BL/6 hosts. Bar = 1 cm. Dot plot shows means ± s.d. of tumor weight. N=5 xenografts. Two-sided unpaired t-test. (k) Image of subcutaneous tumors grown from Pten/Kras cells cocultured with control and Foxf2-expressing mouse prostate stromal cells (mPrSC) in SCID/Beige hosts. Bar = 1 cm. Dot plot shows means ± s.d. of tumor weight. N=8 xenografts. Two-sided unpaired t-test.

Supplementary Figure 3

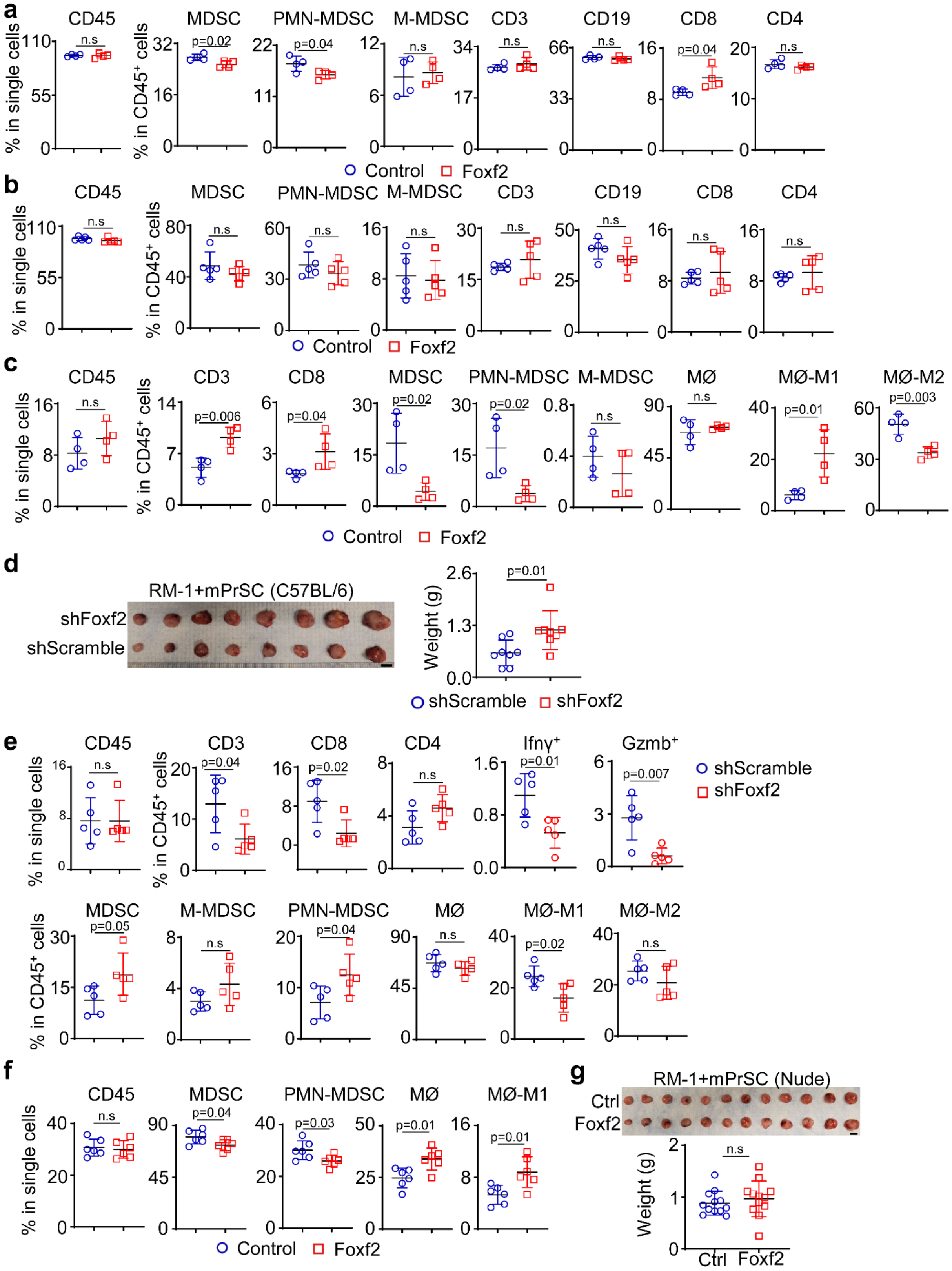

Supplementary Fig. 3: Increasing stromal Foxf2 affects immuno-microenvironment *in vivo*.

(a-b) FACS analyses of immune cells in spleen (a) and blood (b) of C57BL/6 mice harboring subcutaneous RM-1 tumors grown with control and Foxf2-expressing stromal cells. Dot plots show means ± s.d. of cell percentages. N=4 (spleen) or 5 (blood). Two-sided unpaired t-test. (c) FACS analysis of immune cells in Pten/Kras tumors grown with control and Foxf2-expressing stromal cells in C57BL/6 hosts. Dot plots show means ± s.d. of cell percentages. N=4 xenografts per group. Two-sided unpaired t-test. (d) Representative image of RM-1 tumors grown subcutaneously with mouse prostate stromal cells expressing scrambled shRNA (shScramble) or shRNA against *Foxf2* (shFoxf2) in male C57BL/6 hosts. Dot plot shows means ± s.d. of tumor weight. N=8 per group. Two-sided unpaired t-test. Bar = 1 cm. (e) Dot plots show means ± s.d. of percentages of T cells (CD3, CD8, CD4), myeloid derived suppressor cells (M- and PMN-MDSC), and macrophages (MØ, M1 and M2) in RM-1 tumors grown with mouse prostate stromal cells expressing scrambled shRNA (shScramble) or shRNA against *Foxf2* (shFoxf2). N=5 per group. Two-sided unpaired t-test. (f) FACS analysis of immune cells in RM-1 tumors grown with control and Foxf2-expressing stromal cells in SCID/Beige mice. Dot plots show means ± s.d. of cell percentages. N=6 per group. Two-sided unpaired t-test. (g) Image of subcutaneous RM-1 tumors grown with control and Foxf2-expressing mouse prostate stromal cells in male nude mice. Bar= 1 cm. Dot plot shows means ± s.d. of tumor weight. N=12 per group. Two-sided unpaired t-test.

Supplementary Figure 4

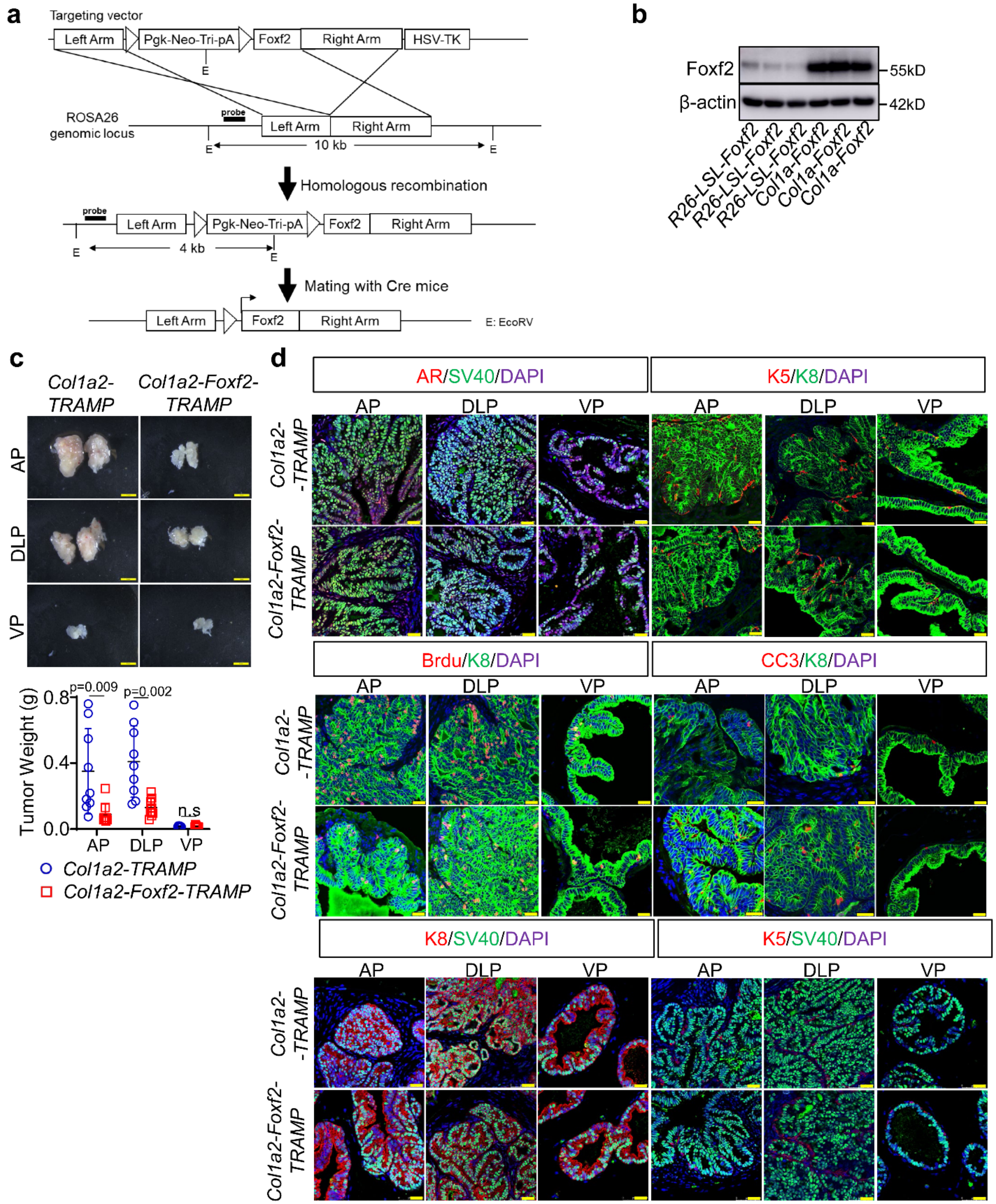

Supplementary Fig. 4: Increasing stromal Foxf2 expression suppresses tumor progression in TRAMP mice.

(a) Schematic illustration of generation of *R26-LSL-Foxf2* model. (b) Western blot analysis of Foxf2 using FACS isolated prostate stromal cells from tamoxifen treated 10-wk-old control (*R26-*

LSL-Foxf2) and *Col1a2-Foxf2* mice. Experiment was done once using 3 different specimens per group shown in the image. Source data are provided in Supplementary Fig. 13. (c) Transillumination images of anterior (AP), ventral (VP), and dorsolateral (DLP) prostates of 9-mth-old control (*Col1a2-TRAMP*) and *Col1a2-Foxf2-TRAMP* mice. Dot plot shows means ± s.d. of prostate weight. N=9 mice for AP and DLP; N=7 for VP. Two-sided unpaired t-test. Bars = 2 mm. (d) Coimmunostaining of AR and SV40 T, K5 and K8, BrdU and K8, and Cleaved caspase 3 (CC3) and K8 in AP, DLP, and VP of 9-mth-old *Col1a2-TRAMP* and *Col1a2-Foxf2-TRAMP* mice. Bars = 25 µm. Experiments were performed on 5 different mice with similar observations.

Supplementary Figure 5

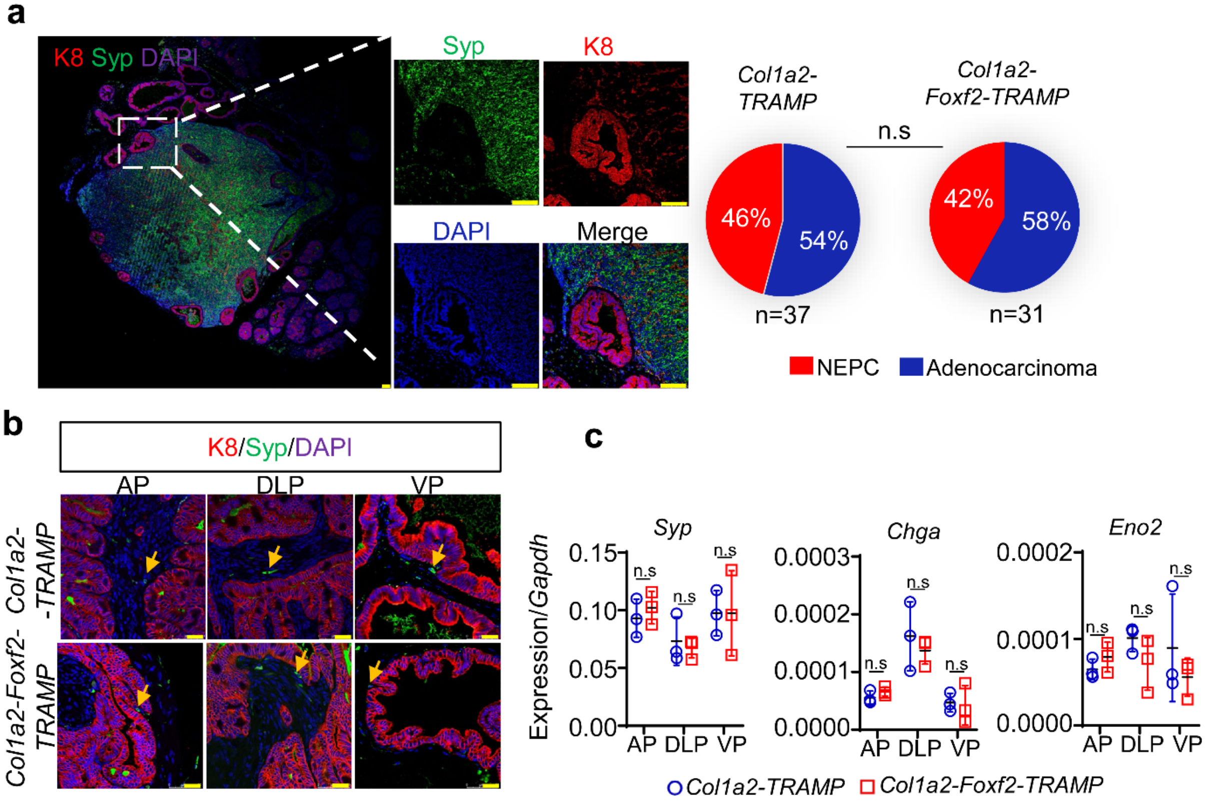

Supplementary Fig. 5: Increasing stromal Foxf2 expression does not affect neuroendocrine prostate cancer (NEPC) frequency in TRAMP mice.

(a) Co-immunostaining of Keratin 8 (K8) ad Synaptophysin (Syp) shows a representative focal NEPC in ventral prostate of a 9-mth-old *TRAMP* mouse. Enlarged region highlight interface between NEPC and adenocarcinoma. Pie charts show quantification of NEPC frequency in 31 *Col1a2-Foxf2-TRAMP* and 37 control *Col1a2-TRAMP* mice. Bars = 100μm. Fischer's exact test. (b) Co-immunostaining of Keratin 8 (K8) and Synaptophysin (Syp) of adenocarcinoma in anterior (AP), dorsolateral (DLP) and ventral (VP) prostates of 9-mth-old *Col1a2-Foxf2-TRAMP* and control *Col1a2-TRAMP* mice. Bars = 25μm. Yellow arrows point to Syp staining. Experiments were performed on 5 different mice with similar observations. (c) QRT-PCR of NEPC-associated genes in adenocarcinoma in anterior (AP), dorsolateral (DLP) and ventral (VP) prostates of 9-mth-old *Col1a2-Foxf2-TRAMP* and control *Col1a2-TRAMP* mice. Data represent means ± s.d. from 3 tissues from different mice. Two-sided unpaired t-test.

Supplementary Figure 6

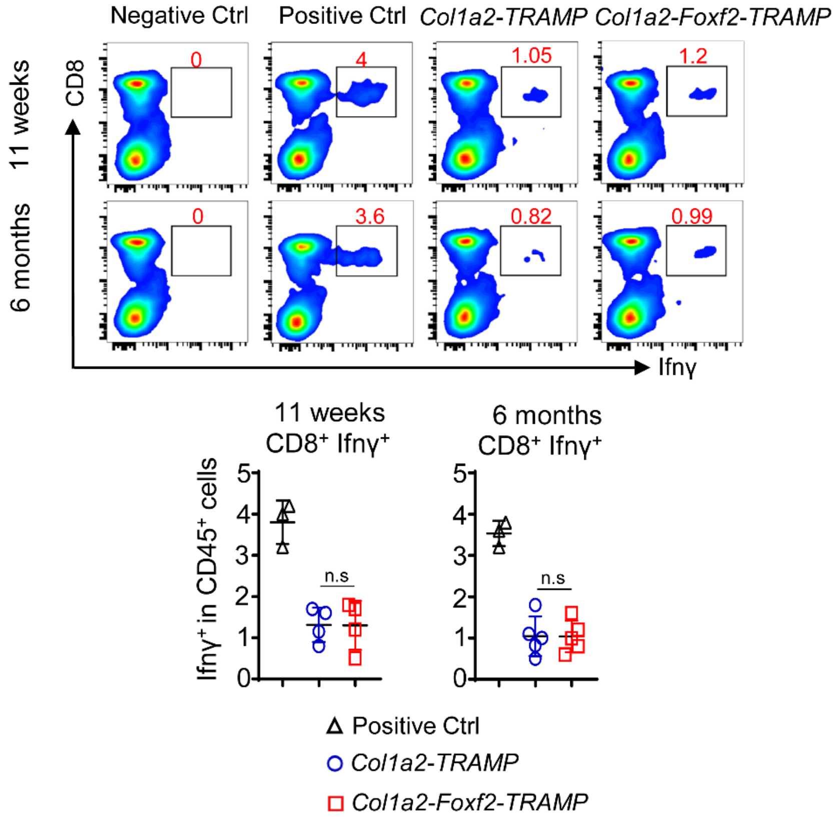

Supplementary Fig. 6: Increasing stromal Foxf2 expression does not affect development of peripheral tolerance toward SV40 Tag in TRAMP mice.

11-wk-old and 6-mon-old *Col1a2-Foxf2-TRAMP* and control *Col1a2-TRAMP* mice were immunized with SV40 Tag-IV. Immunized and unimmunized age-match male C57BL/6 mice served as positive and negative controls, respectively. Splenocytes isolated from these mice were stimulated with SV40 Tag-IV. FACS plots show percentage of IFNγ-expressing $CD8^+$ T cells. Data represent means ± s.d. from 3 mice in positive control, 4 mice per group for 11-wk-old mice and 5 mice per group for 6-mon-old mice. One-way ANOVA with Tukey's multiple comparison test.

Supplementary Figure 7

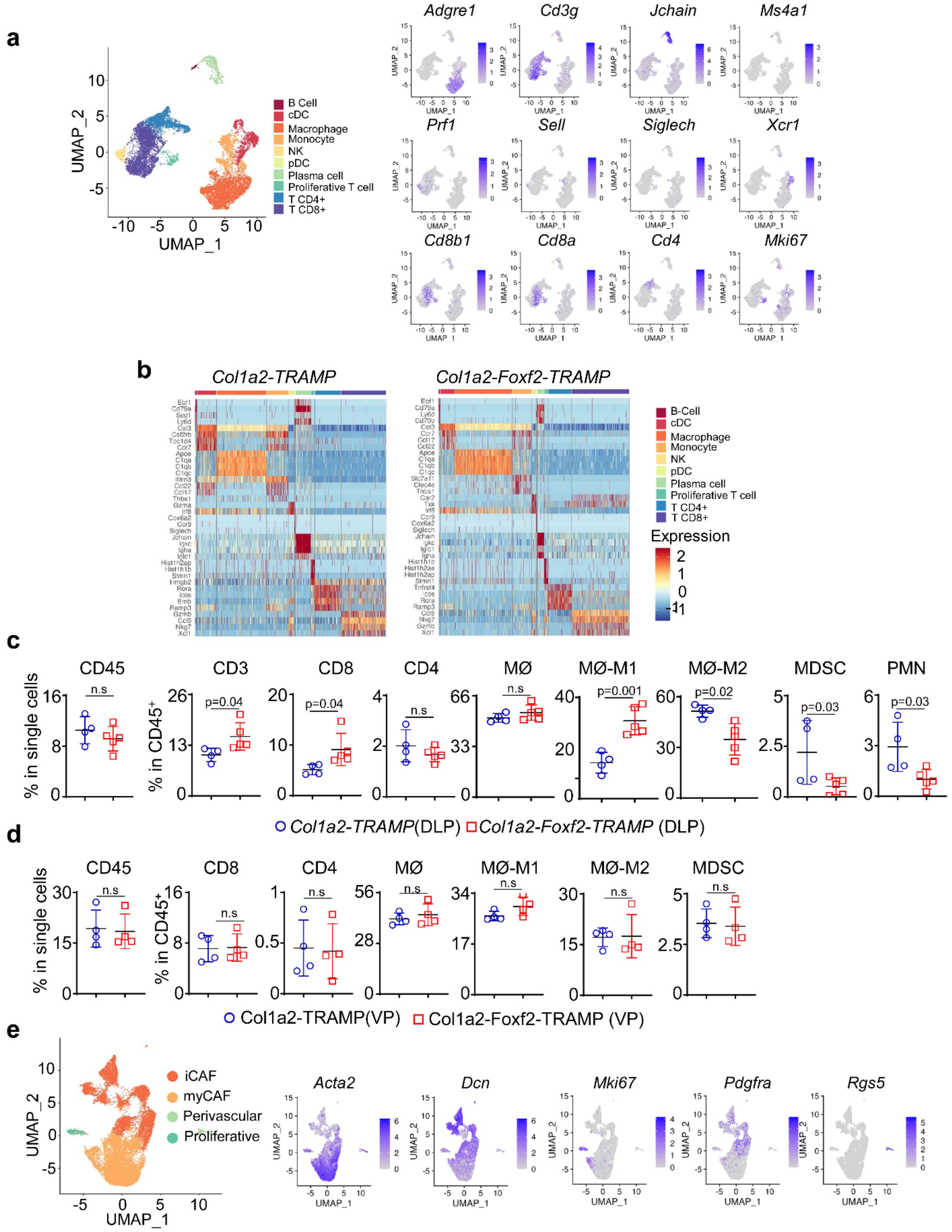

Supplementary Figure 7 continued

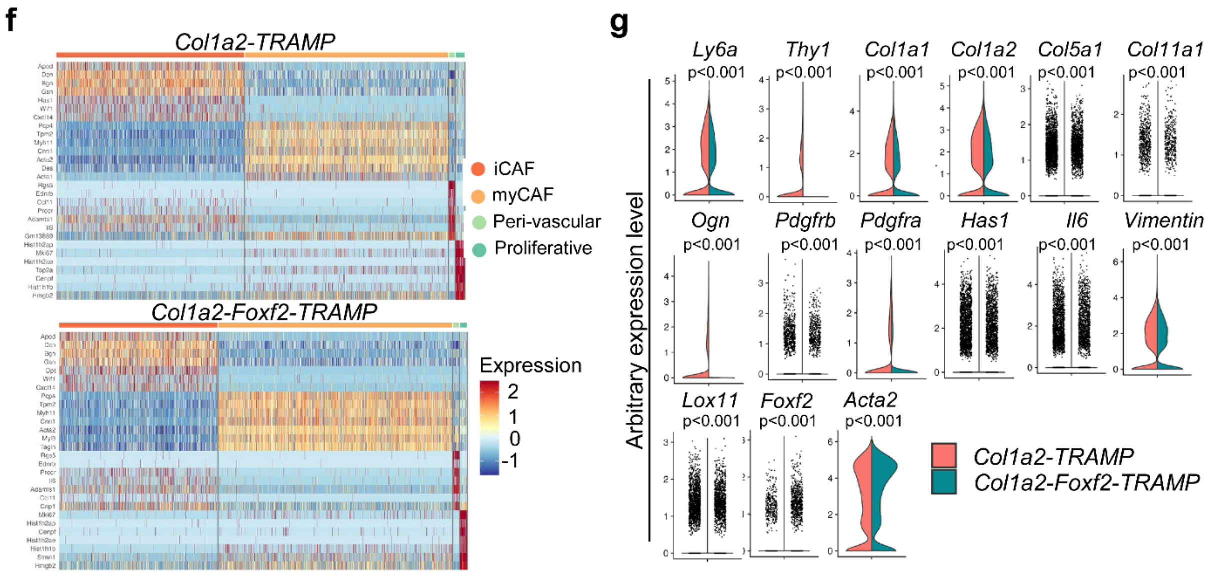

Supplementary Fig. 7: Single cell RNA-seq analysis of immune and stromal cells in *Col1a2-Foxf2-TRAMP* and control *Col1a2-TRAMP* mice.

(a) UMAP plot of FACS isolated CD45$^+$ cells in prostates identifies 10 subpopulations. Panels on the right show expression of representative genes in specific subpopulations. (b) Expression profile heat map of immune cell signature genes for indicated cell clusters. (c) FACS analysis of immune cell subpopulations in dorsolateral prostate cancer tissues of 9-mth-old control (*Col1a2-TRAMP*) and *Col1a2-Foxf2-TRAMP* mice. Data represent means ± s.d. of cell percentages from 4 *Col1a2-TRAMP* and 5 *Col1a2-Foxf2-TRAMP* mice. Two-sided unpaired t-test. (d) FACS analysis of immune cell subpopulations in ventral prostate cancer tissues of 9-mth-old control (*Col1a2-TRAMP*) and *Col1a2-Foxf2-TRAMP* mice. Data represent means ± s.d. of cell percentages from 4 mice per group. Two-sided unpaired t-test. (e) UMAP plot of FACS isolated Lin$^-$CD49f$^-$CD24$^-$ prostate stromal cells identifies 4 subpopulations. Panels on the right show expression of representative genes in specific subpopulations. (f) Expression profile heat map of CAF signature genes for indicated cell clusters. (g) Violin plots show expression of genes related to cancer-associated fibroblasts (CAFs) in single stromal cells isolated from 9-mth-old *Col1a2-TRAMP* and *Col1a2-Foxf2-TRAMP* mice compared using a Wilcoxon Rank Sum test within Seurat's "FindAllMarkers" function to identify differentially expressed genes. Adjusted p-value based on Bonferroni correction using all genes in the dataset.

Supplementary Figure 8

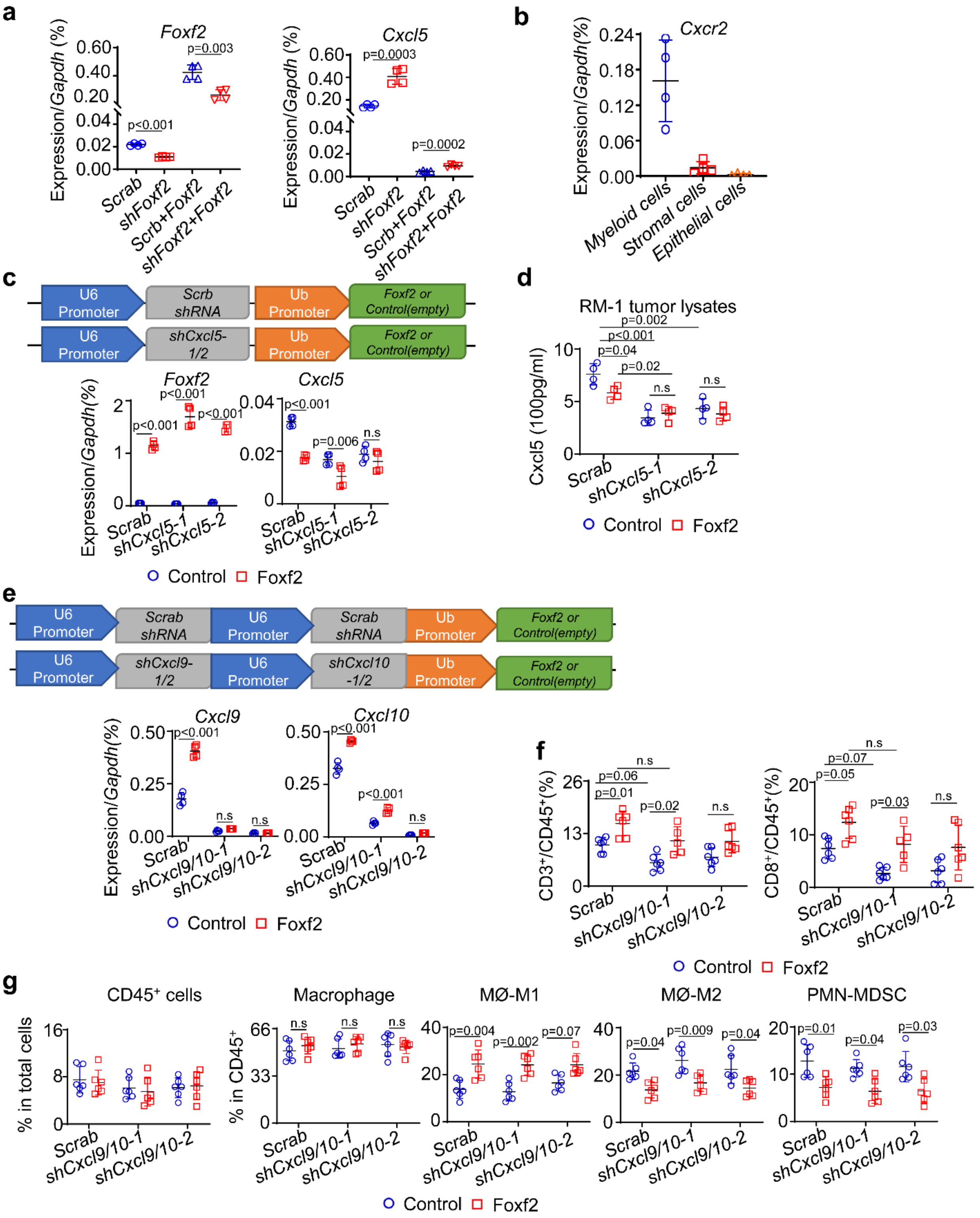

Supplementary Fig. 8: Cxcl5 plays a crucial role in stromal Foxf2-mediated tumor suppression.

(a) QRT-PCR of *Foxf2* and *Cxcl5* in mouse prostate stromal cells infected with lentivirus expressing scrambled or *Foxf2* shRNA with or without ectopically expressed *Foxf2*. Data represent

means ± s.d. of gene expression from 4 independent experiments. Two-sided unpaired t-test. (b) QRT-PCR of *Cxcr2* in FACS-isolated myeloid, stromal, and RM-1 tumor cells from RM-1 xenografts grown subcutaneously in male C57BL/6 hosts. Data represent means ± s.d. of gene expression from 4 independent experiments. (c) QRT-PCR of *Foxf2* and *Cxcl5* in mouse prostate stromal cells infected with lentivirus expressing scrambled or *Cxcl5* shRNA with or without *Foxf2*. Data represent means ± s.d. of gene expression from 4 independent experiments. Two-way ANOVA with Tukey's multiple comparison test. (d) ELISA of Cxcl5 in tumor lysates. Data represent means ± s.d. of protein level from 4 tumors per group. Two-way ANOVA with Tukey's multiple comparison test. (e) qRT-PCR of *Foxf2* and *Cxcl9/10* in mouse prostate stromal cells infected with lentivirus expressing scrambled or *Cxcl9/10* shRNAs with or without *Foxf2*. Data represent means ± s.d. of gene expression from 4 independent experiments. Two-way ANOVA with Tukey's multiple comparison test. (f-g) Dot plots show means ± s.d. of percentages of $CD45^+$, $CD3^+$, $CD8^+$, MDSC, PMN-MDSC, M1- and M2-macrophages cells determined by flow cytometry in RM-1 tumors grown with control and Foxf2-expressing stromal cells with scrambled or *Cxcl9/10* shRNAs. N= 5 to 6 per group. Two-way ANOVA with Tukey's multiple comparison test.

Supplementary Figure 9

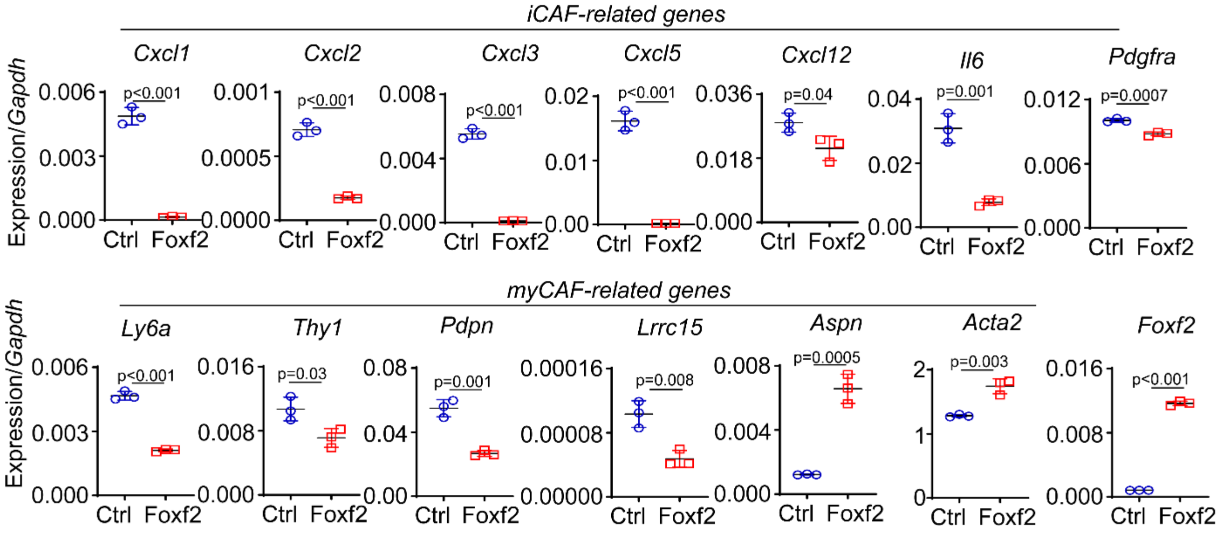

Supplementary Fig. 9: Stromal Foxf2 expression affects CAF phenotype.
QRT-PCR of 13 CAF-associated genes in control and Foxf2-expressing mouse prostate stromal cells. Data represent means ± s.d. from 3 experiments. Two-sided unpaired t-test.

Supplementary Figure 10

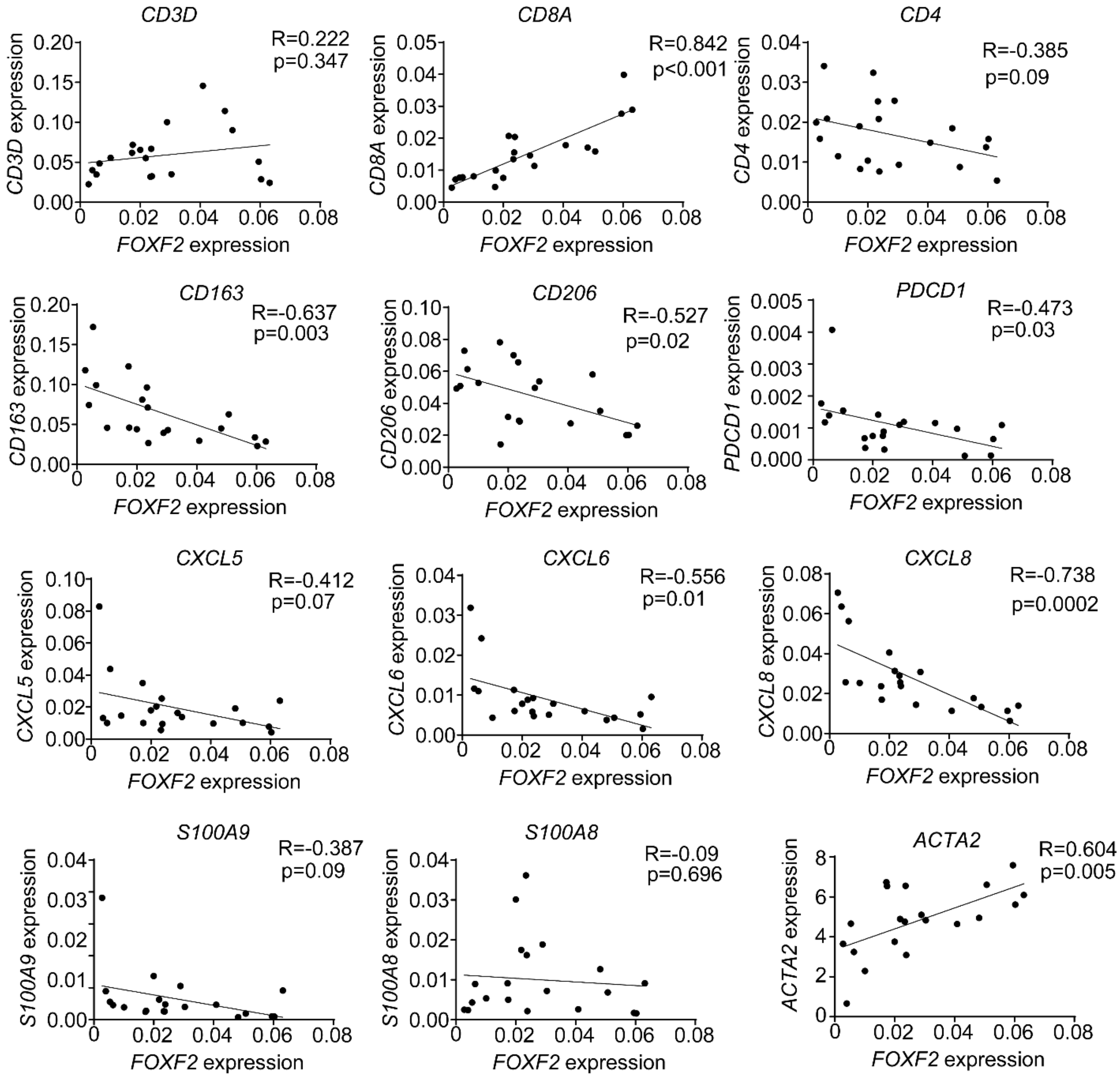

Supplementary Fig. 10: Correlation between expression of *FOXF2* with those of immune cell markers in human prostate cancer specimens.

Each dot represent result obtained by qRT-PCR analysis from one laser-captured stromal region in human prostate cancer specimen. A total of 20 regions were collected from 13 different patient specimens including benign and cancerous tissues with Gleason patterns ranging from 3 to 5. Data analyzed by Pearson correlation analysis.

Supplementary Figure 11

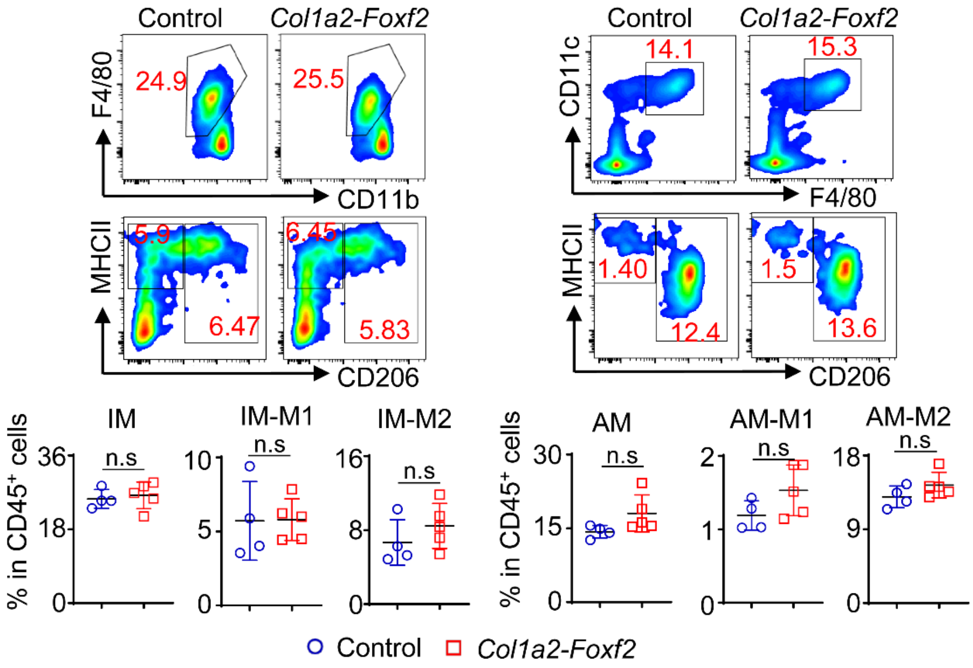

Supplementary Fig. 11: Increasing lung stromal Foxf2 suppresses prostate cancer metastasis. FACS plots of pro-inflammatory (M1) and anti-inflammatory (M2) infiltrating macrophage (IM) and alveolar macrophage (AM) in lung metastases. Dot plots show means ± s.d. of cell percentages from 4 (control) or 5 (Col1a2-Foxf2) tumors. Two-sided unpaired t-test.

Supplementary Figure 12

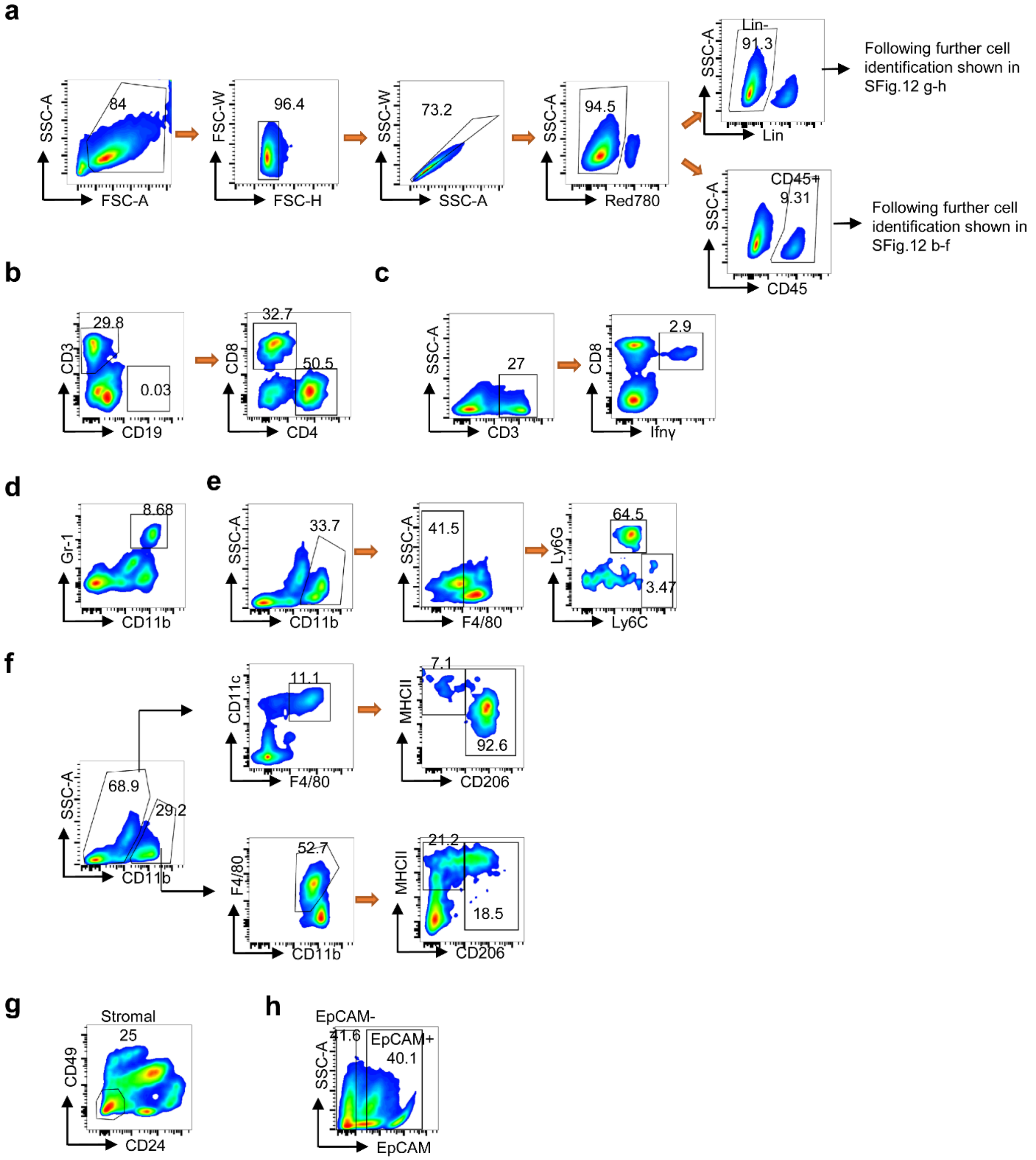

Supplementary Fig. 12: Illustration of gating strategy for flow cytometric analyses and sorting.

(a) Single viable Lineage^{+} or CD45^{+} cells. (b) CD4 and CD8. (c) Ifnγ. (d) CD11b and Gr-1. (e) Ly6C and Ly6G. (f) CD206 and MHCII. (g) CD24 and CD49 staining of mouse prostate lineages. (h) EpCAM staining of cells in RM-1 xenograft.

Supplementary Fig. 13: Source data for Western Blot analyses in Supplementary Figs. 1A, 2C and 3B.

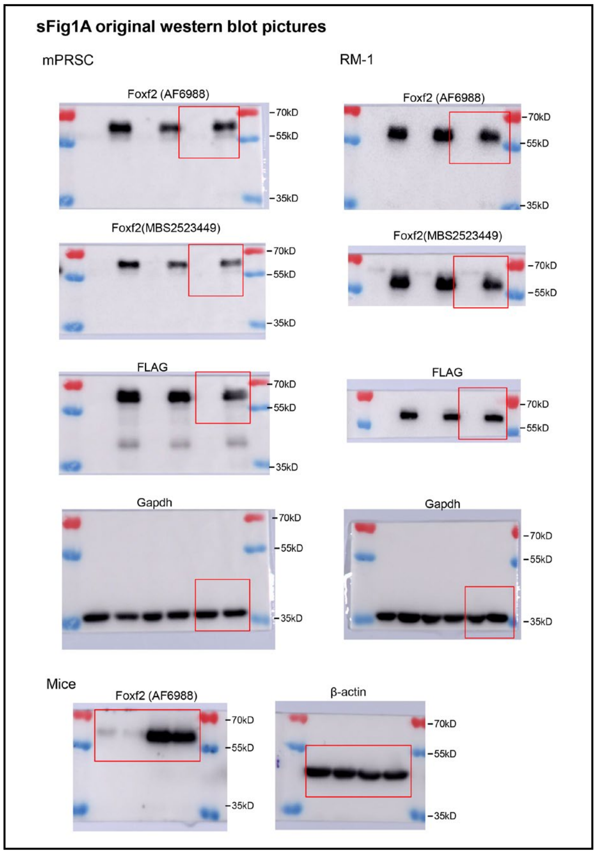

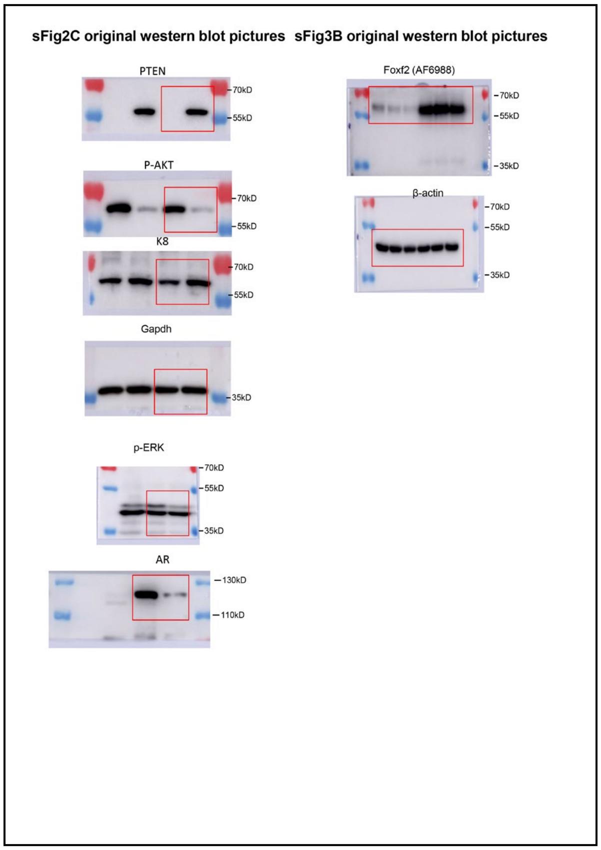

Supplementary Table 1: Cytokine/Chemokine expression levels in Primary stromal cells(pg/ml)

Cytokine/ Chemokine	Means ± SD (n=3)		p-value, (unpaired t-test)
	Primary stromal cells (Vector)	Primary stromal cells (Foxf2)	
CCL11	18.23+1.3	42.79+7.08	0.0041
GM-CSF	9.42+0.73	6.75+2.1	0.1058
IL-1a	5.3+3.5	4.6+2.42	0.7907
IL-15	6.07+3.28	5.35+3.88	0.8190
LIF	88.6+4.96	9925+6.74	0.0923
G-CSF	4.88+1.23	5.27+0.7	0.6575
IFNy	2.43+0.42	1.41+0.76	0.1134
IL-1B	6.77+2.54	9.07+1.91	0.2781
MIP-1a (CCL3)	58.18+10.85	29.18+10.02	0.0272
MCP-1	3689.72+369.62	4665.73+309.19	0.0247
IL-2	2.78+1.49	2.08+0.41	0.4773
IL-6	2812.77+99.49	1893.88+179.13	0.0015
IL-9	15.24+3.95	13.24+4.82	0.6082
KC (CXCL1)	162.54+34.33	138.84+12.35	0.3236
M-CSF (CSF1)	31.22+2.55	27.69+2.35	0.1524
TNFa	3.59+0.23	2.02+0.26	0.0014
VEGF	468.23+45.84	718.54+94.61	0.0146
MIP-2 (CXCL2)	44.02+13.34	38.18+15.33	0.6449
RANTES (CCL5)	50.5+3.86	40.67+2.42	0.0201
MIP-1b (CCL4)	96.24+17.23	43.17+8.33	0.0086

LIX (CXCL5)	39.59+12.14	8.34+2.91	0.0087
MIG (CXCL9)	2.27+0.3	3.21+0.21	0.0043
IP-10 (CXCL10)	197.01+9.61	261.45+45.23	0.0305

Cytokine/Chemokine expression levels in tumor samples(pg/ml)

Cytokine/ Chemokine	Means ± SD (n=3)		p-value (unpaired t-test)
	Tumor samples-Vector	Tumor samples-Foxf2	
CCL11	263.22+137.29	349.66+91.35	0.1603
GM-CSF	208.13+145.76	178.28+147.11	0.6897
IL-1a	92.40+25.84	109.91+41.51	0.3286
IL-15	38.82+10.77	67.06+22.11	0.0058
LIF	239.55+44.14	317.76+59.32	0.0097
G-CSF	971.28+230.6	999.62+487.98	0.8840
IFNy	15.26+8.99	38.22+28.26	0.0460
IL-1B	21.82+9.27	19.38+6.66	0.5553
MIP-1a (CCL3)	117.45+26.97	192.87+111.61	0.1078
MCP-1	6030.58+1873.84	7701.85+2835.69	0.1860
IL-2	15.59+3.19	16.11+4.22	0.7850
IL-6	48.11+25.65	24.69+7.25	0.0262
IL-9	77.80+22.47	71.49+9.38	0.4758
KC (CXCL1)	1456.16+748.39	1472.70+1172.77	0.9737
M-CSF (CSF1)	18.50+9.46	22.44+10.32	0.4387
TNFa	3.51+0.91	5.78+2.37	0.0243
VEGF	701.87+246.47	677.90+355.67	0.8778

MIP-2 (CXCL2)	5721.70+4587.96	5383.09+5141.65	0.8914
RANTES (CCL5)	17.62+10.03	33.57+18.97	0.0541
MIP-1b (CCL4)	66.58+28.22	131.93+96.49	0.0873
LIX (CXCL5)	666.95+188.51	510.27+108.05	0.0460
MIG (CXCL9)	829.01+257.34	1705.39+712.82	0.0099
IP-10 (CXCL10)	540.70+190.14	1534.17+823.16	0.0090

Supplementary Table 2: Genotyping Primers and expected band size

Primers	Sequences (5' to 3')	WT(bp)	TG(bp)
Cre forward	CATTTGGGCCAGCTAAACAT	308	N/A
Cre reverse	ATTCTCCCACCGTCAGTACG		
Tramp Wild type forward	CTG TAC CAA TGG GCT TGA GG	264	151
Tramp common reverse	GGC ATT CAG CTT GGA GAA GA		
Tramp Mutant forward	TCA CAA ATT TCA CAA ATA AAG CA		
Foxf2 Wild type forward	TCCCAAAGTCGCTCTGAGTT	219	300
Foxf2 Wild type reverse	TAAGCCTGCCCAGAAGACTC		
Foxf2 ^{lox} forward	CCTACTGGTTGGAGCAGAGC		
Foxf2 ^{lox} reverse	GCGATGCAATTTCCTCATTT		
eYFP forward	CTCTGCTGCCTCCTGGCTTCT	330	250
eYFP reverse 1	CGAGGCGGATCACAAGCAATA		
eYFP reverse 2	TCAATGGGCGGGGGTCGTT		

Supplementary Table 3: Antibodies for Flow Cytometry or Sorting cells

Antigen	Supplier	Cone number	Cat. Number	Dilution
CD45-FITC	Ebioscience	30-F11	11-0451-85	1:100
CD8- PECY7	Ebioscience	53-6.7	25-0081-82	1:100
Ly6C-PE	Ebioscience	HK1.4	12-5932-82	1:100
GR-1-APC	Ebioscience	RB6-8C5	17-5931-82	1:100
F4/80-PECY7	Ebioscience	BM8	25-4801-82	1:100
CD11B- eFluor 710	Ebioscience	M1/70	48-0112-82	1:100
Granzyme B-Pacific blue	Biolegend	GB11	515407	1:100
IFN-γ-PE	Biolegend	XMG1.2	505808	1:100
CD11C-PE	Biolegend	N418	117308	1:100
CD3-PE	Ebioscience	145-2C11	12-0031-83	1:100
CD3-APC	Biolegend	145-2C11	100312	1:100
CD4-PE	Biolegend	GK1.5	100408	1:100
CD206-APC	Ebioscience	MR6F3	17-2061-82	1:100
MHCII-PE	Biolegend	M5/114.15.2	107608	1:100
CD19-FITC	Biolegend	6D5	115506	1:100
MHCII-Pecy5.5	Biolegend	M5/114.15.2	107611	1:100
TNFα-FITC	Biolegend	MP6-XT22	506304	1:100
Ly-6G- PerCP-eFluor 710	Ebioscience	1A8-Ly6g	46-9668-82	1:100
IFNγ-APC	Biolegend	XMG1.2	505810	1:100
CD24-FITC	Ebioscience	M1/69	11-0242-85	1:100

CD49-APC	Ebioscience	GoH3	17-0495-82	1:100
CD45- eFluor 450	Ebioscience	30-F11	48-0451-82	1:100
CD31- eFluor 450	Ebioscience	390	48-0311-82	1:100
TER119- eFluor 450	Ebioscience	TER-119	48-5921-82	1:100
EpCAM(CD326)-PECY7	Biolegend	G8.8	118216	1:100
Ghost Dye Red 780	Cell Signaling		18452	1:1000

Supplementary Table 4: mouse primers for qRT-PCR analysis

Primers	Sequences (5' to 3')
Gapdh forward	TGTTCCTACCCCCAATGTGT
Gapdh reverse	GGTCCTCAGTGTAGCCCAAG
Foxf2 forward	CCTACTCGTTGGAGCAGAGC
Foxf2 reverse	GGAGTGGAGTGGTGCTGGTA
Cxcl5 forward	TGCATTCCGCTTAGCTTTCT
Cxcl5 reverse	CAGAAGGAGGTCTGTCTGGA
Cxcl9 forward	GGAGTTCGAGGAACCCTAGTG
Cxcl9 reverse	GGGATTTGTAGTGGATCGTGC
Cxcl10 forward	CCAAGTGCTGCCGTCATTTTC
Cxcl10 reverse	GGCTCGCAGGGATGATTTCAA
Cd8a forward	GACATCTCAGCCCCAGAGAC
Cd8a reverse	CAAGGGTGCCCAGATGTAAA
Lck forward	CGTGTGTGAAAACTGCCACT
Lck reverse	GAGATCCCTCATAGGTGACCAG
Syk forward	TCCAGAGATGAATCAGAGCAGA
Syk reverse	GCAGGCACAGAGCATAGGAG
Gzmb forward	ATGTGGGGGCTTCCTTATTC
Gzmb reverse	TTGCTGGGTCTTCTCCTGTT
Ncf1 forward	ACACCTTCATTCGCCATATTGC
Ncf1 reverse	TCGGTGAATTTTCTGTAGACCAC
Ncf4 forward	GTGAACTCGGCCTGGATCTG
Ncf4 reverse	AAGCTGCTCAAAGTCGCTCT

Cybb forward	CCTCTACCAAAACCATTCGGAG
Cybb reverse	CTGTCCACGTACAATTCGTTCA
Il10 forward	ATCGATTTCTCCCCTGTGAA
Il10 reverse	TGTCAAATTCATTCATGGCCT
Arg1 forward	TTTTTCCAGCAGACCAGCTT
Arg1 reverse	AGAGATTATCGGAGCGCCTT
iNOS forward	TTCTGTGCTGTCCCAGTGAG
iNOS reverse	TGAAGAAAACCCCTTGTGCT
Ifng forward	CGGCACAGTCATTGAAAGCCTA
Ifng reverse	GTTGCTGATGGCCTGATTGTC
Tgfb1 forward	ATTCAGCGCTCACTGCTCTT
Tgfb1 reverse	GGTTCATGTCATGGATGGTG
Tnfa forward	CCACCACGCTCTTCTGTCTA
Tnfa reverse	AGGGTCTGGGCCATAGAACT
Cxcl1 forward	ACTGCACCCAAACCGAAGTC
Cxcl1 reverse	TGGGGACACCTTTTAGCATCTT
Cxcl2 forward	CCAACCACCAGGCTACAGG
Cxcl2 reverse	GCGTCACACTCAAGCTCTG
Cxcl3 forward	GATTTTGAGACCATCCAGAGC
Cxcl3 reverse	CTCTTCAGTATCTTCTTGATG
Cxcl12 forward	GTAAACCAGTCAGCCTGAG
Cxcl12 reverse	GCTTTCTCCAGGTACTCTTG
Il6 forward	TCACAAGTCGGAGGCTTAAT
Il6 reverse	TTCTGCAAGTGCATCATCGT

Pdgfrα forward	ACGGATGAGAGTGAGATCGAA
Pdgfrα reverse	CGATGACTAAGGAATCGGTCA
Acta2 forward	TGGAAGAAGGAAACCTGGAA
Acta2 reverse	GGAACTGCACACTGCTGGTA
Ly6a forward	CCATCAATTACCTGCCCCTA
Ly6a reverse	GGCAGATGGGTAAGCAAAGA
Thy1 forward	GGCGACTACTTTTGTGAGCTT
Thy1 reverse	GGAGGAGGGAGGGGGAAAG
Pdpn forward	CAACCACAGGTGCTACTGGA
Pdpn reverse	GCTGAGGTGGACAGTTCCTC
Lrrc15 forward	CTGCCCTGGAATGCTATGAG
Lrrc15 reverse	CCAGCTCGTTCTTCTCCATC
Aspn forward	TTGGGTGCCAATGTTACTCTC
Aspn reverse	TTGAAGGTCAACCATTCGAGTA
Cxcr2 forward	CCTTCTACAGCAGTGTTCTGCTAC
Cxcr2 reverse	TTGACCAAGTGTCTCTTCTGGA
Aldh1a1 forward	CCTGCAACTGAGGAGGTCAT
Aldh1a1 reverse	TGGAGAGCCAATCTGGAAAG
Pou5f1 forward	GCTGGAGAAGTGGGTGGAG
Pou5f1 reverse	TGCTAGTTCGCTTTCTCTTCC
Syp forward	TGGTGTCAATAAGAACCAGACTGT
Syp reverse	TCCCAGTTCACGTCACACAC
Chga forward	CAGAAGTGTTTGAGAACCAGAGC
Chga reverse	TTGTCAGAATCCTTTCTCTTCTCC

Eno2 forward	TGGACAACCTGATGTTGGAA
Eno2 reverse	CTGCCCCAGCCTTACACAC
Vim forward	GAGGTGGAGCGGGACAAC
Vim reverse	GAATGACTGCAGGGTGCTTT
Twist2 forward	GCAAGAAATCGAGCGAAGAT
Twist2 reverse	AGGCCTCGTTGAGCGACT
Slug forward	AACATTTCAACGCCTCCAAG
Slug reverse	TCTCTGGTTTTGGTATGACAGG
Zeb1 forward	TTACCAGGAGGCAGTGACAG
Zeb1 reverse	CCACATTCGGATCATGGTTT
Snail1 forward	TGGAAAGGCCTTCTCTAGGC
Snail1 reverse	GTCAGCAAAAGCACGGTTG
Mmp2 forward	AACTTCTTCCCCCGCAAG
Mmp2 reverse	AAAGCATCATCCACGGTTTC
Cd44 forward	CATCAGTCACAGACCTACCCAAT
Cd44 reverse	GTTCTATACTCGCCCTTCTTGC
Myc forward	AGTGCTGCATGAGGAGACAC
Myc reverse	GGTTTGCCTCTTCTCCACAG

Supplementary Table 5: Human primers for qRT-PCR analysis

Primers	Sequences (5’ to 3’)
GAPDH forward	ACGGGAAGCTTGTCATCAAT
GAPDH reverse	TGGACTCCACGACGTACTCA
S18 forward	TGAGGAAAGCAGACATTGACC
S18 reverse	TTGTACTGGCGTGGATTCTG
FOXF2 forward	ACTCGCTGGAGCAGAGCTAC
FOXF2 reverse	GGACGAAATCTTTTCTGTCACAC
CD3D forward	CTGCCGACACACAAGCTCT
CD3D reverse	GTTCCGAGCCCAGTTTCCT
CD8A forward	ACTTGTGGGGTCCTTCTCCT
CD8A reverse	GTCTCCCGATTTGACCACAG
CD4 forward	ATGCACTGTCTTGCAGAACC
CD4 reverse	CACCTGTTCCCCCTCTTTCT
CD163 forward	GCTCTAGGTGCTTCATTATGTCC
CD163 reverse	CCAAAGACGATGAATTGCAC
CD206 forward	TGCAGAAGCAAACCAAACCT
CD206 reverse	AGGCCTTAAGCCAACGAAAC
PDCD1 forward	CGCACGAGGGACAATAGG
PDCD1 reverse	GGAAATCCAGCTCCCCATAG
CXCL5 forward	CTGTTGGTGCTGCTGCTG
CXCL5 reverse	GGTCTGTAAACAAACGCAACG
CXCL6 forward	GCTGCTGCTCCTGCTGAC
CXCL6 reverse	TCAGCGTAACGCGTAAACA

CXCL8 forward	GAACTGAGAGTGATTGAGAGTGGA
CXCL8 reverse	CTCTGCACCCAGTTTTCCTT
S100A8 forward	CGTCTACAGGGATGACCTGA
S100A8 reverse	AACTGCACCATCAGTGTTGA
S100A9 forward	ACCAGGGGGAATTCAAAGAG
S100A9 reverse	GTCCAGGTCCTCCATGATGT
ACTA2 forward	CCGACCGAATGCAGAAG GA
ACTA2 reverse	ACAGAGTATTTGCGCTCCGAA

Supplementary Table 6: qPCR Primers for ChIP assay

Primers	Sequences (5' to 3')
Chr.15 forward	AGCGTGGCCTTGGCAGCAAA
Chr.15 reverse	TGCGATTGGCTTCCTCTCCCC
mPDGFα forward	CCCATCATCCTCTCCTCTTTGC
mPDGFα reverse	GGTAGCCTTTAGAAATGTGTGCCAG
CXCL5 site1 forward	TTCTTTCTTTCTTTCTTTCTTTCTTTC
CXCL5 site1 reverse	GGAAATGTAATCCCGGACTTG
CXCL5 site2 forward	GTCCGGGATTACATTTCCTG
CXCL5 site2 reverse	GATAAGCAGACATACACATGCTCA

Supplementary Table 7: Primers for cloning CXCL5 Luciferase Reporters

Primers	Sequences (5' to 3')
Foxf2 Site 1 mutagenesis F	TTTTTATCTTTTCAAGTCCGG
Foxf2 Site 1 mutagenesis R	AAAAGAAAGAAAGAAAGAAAGAAAGAAAG
Foxf2 Site 2 mutagenesis F	TTTGAGCATGTGTATGTCTGC
Foxf2 Site 2 mutagenesis R	AAAAAAAATACATTTGAAATGTGTGTTTTTAAAAC
NFκB Site deletion F	GCATCTTCAAGCTCCGCT
NFκB Site deletion R	GACTTGGAGTTAGGAGGTG

Supplementary Table 8: Antibodies for IHC and Western Blot

Antigen	Supplier	Cat. Number/Clone Number	Species	Dilution
AR	Santa Cruz	sc-816	Rabbit	1:1000
AR	Santa Cruz	sc-13062	Rabbit	1:200
K5	905501	Biolegend	Rabbit	1:1000
K8	Covance	MMS-162P/1E8	Mouse	1:1000
K8	Abcam	AB53280	Rabbit	1:500
CD31	Abcam	AB28364	Rabbit	1:300
GFP	Abcam	ab13970	Chicken	1:1000
BrdU	Abcam	ab6326	Rat	1:500
Cleaved Caspase 3	Cell Signaling	9661S	Rabbit	1:1000
SV40 T-antigen	Abcam	ab16879/PAb416	Mouse	1:300
Smooth Muscle Actin	Sigma	202M/1A4	Mouse	1:1000
Synaptophysin	Novocastra	*NCL*-L-*SYNAP-299/* 27G12	Mouse	1:300
Flag	Sigma	F1804/M2	Mouse	1:1000
Foxf2	Abcam	ab198283	Rabbit	1:500
Foxf2	R&D	AF6988	Sheep	1:100
Foxf2	MyBioSource	MBS2523449	Rabbit	1:500
CTLA-4	Bio X Cell	BE0131	Hamster	200ug
Hamster IgG Isotype	Bio X Cell	BE0087	Hamster	200ug
PD-1	Bio X Cell	BE0146	Rat	200ug
rat IgG2a isotype	Bio X Cell	BE0089	Rat	200ug

β-actin	Sigma	A2228/AC-74	Mouse	1:1000
GAPDH	Cell Signaling	2118	Rabbit	1:1000
normal rabbit IgG	Santa Cruz	sc-2027	Rabbit	1:30
normal donkey Serum	Jackson Imm	017-000-121	Donkey	1:30
PTEN	Cell Signaling	9556/26H9	Mouse	1:1000
p-AKT	Cell Signaling	4060	Rabbit	1:1000
p-Erk1/2	Cell Signaling	4370	Rabbit	1:1000
p-P65	Invitrogen	MA5-15160/T.849.2	Rabbit	1:1000
P65	Cell Signaling	8242	Rabbit	1:1000
p-Stat3	Cell Signaling	4113	Rabbit	1:1000
Stat3	Cell Signaling	8768	Rabbit	1:1000
α-Mouse IgG Alexa Fluor 594	Life technologies	A11020	Goat	1:500
α-Mouse IgG Alexa Fluor 488	Life technologies	A11029	Goat	1:500
α-Rabbit IgG Alexa Fluor 594	Life technologies	A11037	Goat	1:500
α-Rat IgG Alexa Fluor 594	Life technologies	A11006	Goat	1:500
α-Sheep IgG Alexa Fluor 488	Life technologies	A11015	Donkey	1:1500
α-Rabbit HRP	Vector Lab.	PI-1000	Goat	1:5000
α-Mouse HRP	Vector Lab.	PI-2000	Goat	1:5000
α-Sheep IgG HRP	Invitrogen	A16041	Donkey	1:5000
